# Machine Learning Estimation of Plateau Stress of Aluminum Foam Using X-ray Computed Tomography Images

**DOI:** 10.3390/ma16051894

**Published:** 2023-02-24

**Authors:** Yoshihiko Hangai, So Ozawa, Kenji Okada, Yuuki Tanaka, Kenji Amagai, Ryosuke Suzuki

**Affiliations:** Graduate School of Science and Technology, Gunma University, Kiryu 376-8515, Japan

**Keywords:** cellular materials, machine learning, deep learning, neural network, foam, X-ray computed tomography (CT), mechanical properties, plateau stress

## Abstract

Owing to its lightweight and excellent shock-absorbing properties, aluminum foam is used in automotive parts and construction materials. If a nondestructive quality assurance method can be established, the application of aluminum foam will be further expanded. In this study, we attempted to estimate the plateau stress of aluminum foam via machine learning (deep learning) using X-ray computed tomography (CT) images of aluminum foam. The plateau stresses estimated by machine learning and those actually obtained using the compression test were almost identical. Consequently, it was shown that plateau stress can be estimated by training using the two-dimensional cross-sectional images obtained nondestructively via X-ray CT imaging.

## 1. Introduction

Owing to its lightweight and excellent shock-absorbing properties, aluminum foam is used in automotive parts, construction materials, and train parts [1,2,3,4,5]. However, even when aluminum foam products are fabricated using the same manufacturing process and under the same manufacturing conditions, the foaming process results in variations in properties, making it difficult to guarantee quality. If a nondestructive quality assurance method can be established, the application of aluminum foam will be further expanded. The technology needed to observe aluminum foam by X-ray computed tomography (CT) has been developed [6,7,8,9,10,11,12], and there is a possibility that it can be used for the nondestructive quality assurance method for aluminum foam. Currently, aluminum foam properties are generally estimated on the basis of density (porosity, which is the ratio of pore volume to total sample volume) [2,4,13]. However, some studies have shown that even when porosity is the same, differences in pore structural characteristics, such as pore size and shape, and their distribution result in different properties [4,14,15,16,17,18,19]. In addition, finite element analysis is used to evaluate properties by simulations [20,21,22,23]. In particular, image-based finite element analysis using X-ray CT images has been performed to reproduce the compressive properties of aluminum foam [6,24,25,26,27,28,29,30,31]. However, because of the high computational cost of elastoplastic analysis, it is impractical to analyze all aluminum foam products at the time of their manufacture for quality assurance purposes.

In recent years, machine learning has been used to evaluate the mechanical properties of materials and structures, and it has also been applied to porous materials, such as foamed concrete, porous metals, and polystyrene foams [32,33,34,35,36,37]. However, most studies on machine learning use parameters during fabrication or certain features such as the density of the obtained specimen for evaluation. It is considered that there are factors other than the fabrication parameters and features of interest that have some effects on the expression of mechanical properties. Therefore, it would be possible to develop a more accurate prediction method for mechanical properties without determining those fabrication parameters and features. In recent years, the machine learning (deep learning) of images has been studied extensively and applied to many fields, such as medical, biological, architectural, and optical fields [38,39,40,41,42,43], but it has not been applied to porous materials. If the properties of aluminum foam can be predicted directly from X-ray CT images via machine learning, it is expected that quality assurance can be performed rapidly, nondestructively, and accurately at the manufacturing line.

In our previous study [44], it was shown that high-strength aluminum foam (mean plateau stress, 64.2 MPa; mean porosity, 49.4%) and low-strength aluminum foam (mean plateau stress, 36.1 MPa; mean porosity, 70.9%) can be distinguished with an accuracy of more than 95% from two-dimensional cross-sectional X-ray CT images. Here, plateau stress was defined as the average *σ* at *ε* = 20–30% during the compression test of aluminum foam on the basis of Japanese Industrial Standards JIS-H-7902: “Method for compressive test of porous metals” [45]. In this study, we investigated whether plateau stress can be predicted by machine learning from two-dimensional cross-sectional X-ray CT images.

## 2. Materials and Methods

**Figure 1:**
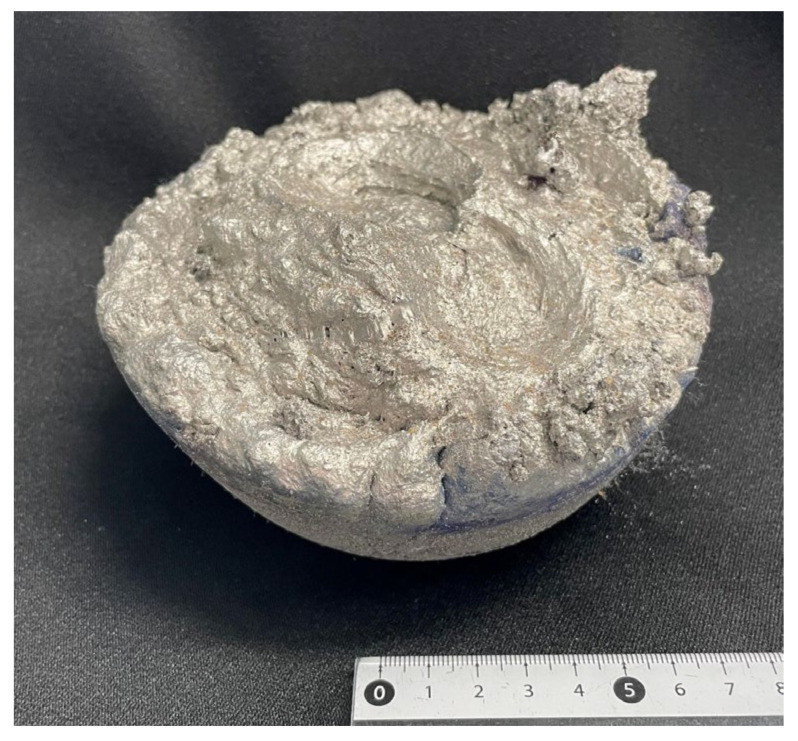
As-fabricated aluminum foam prepared by molten metal foaming method.

The aluminum foam used in this study was fabricated by the molten metal foaming method [46,47,48,49,50,51,52]. Details of the fabrication method can be found in ref. [53]. Briefly, an Al-Si-Cu ADC12 aluminum alloy was used as the base material. The liquidus and solidus temperatures of ADC12 aluminum alloy are 580 °C and 515 °C, respectively [54]. First, ADC12 aluminum alloy was melted in a preheated electric furnace maintained at 800 °C. After the melted ADC12 aluminum alloy was taken out of the furnace, a thickener agent was added and stirred. Then, a blowing agent was added when the molten ADC12 aluminum alloy reached 620 °C, which was measured with a K-type thermocouple and stirred for 30 s before cooling in ambient air. Titanium hydride (TiH_2_, particle size less than 45 μm, 1.5 mass%, Kojundo Chemical Laboratory Co., Ltd., Sakado, Japan) was used as the blowing agent and a hollow ceramic, E-SPHERES SL300 (average particle size of 175 μm, density of 0.85 g/cm^3^, 10 mass%, Taiheiyo Cement Corporation, Tokyo, Japan) was used as the thickener agent. Figure 1 shows a representative sample of as-fabricated aluminum foam. Seven of these samples were prepared. Fourteen specimens for the compression test (cube-shaped, 25 mm each side) were cut from the fabricated aluminum foam samples using an electrical discharge machine. The size of each specimen was determined so that approximately 10 pores could be included on each side with reference to JIS-H-7902 [45] and the pores could be observed with a certain degree of accuracy by X-ray CT imaging used in this study.

**Figure 2:**
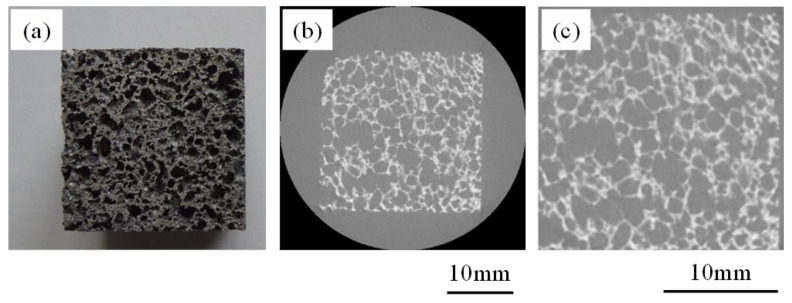
(**a**) Aluminum foam specimen for compression test, (**b**) X-ray CT image of (**a**), and (**c**) image after image processing of (**b**).

The specimens prepared for the compression test were examined by microfocus X-ray CT (SMX-225CT, Shimadzu Corporation, Kyoto, Japan) to observe the internal pore structures. The X-ray source was tungsten in this system. The tube voltage and current were 80 kV and 30 μm, respectively. A cone-type CT system was employed. Figure 2a shows an example of a specimen for the compression test and Figure 2b shows a two-dimensional cross-sectional X-ray CT image of the specimen. In the X-ray CT image, the white areas are aluminum alloy and the gray areas are pores. Three hundred two-dimensional cross-sectional images were obtained for each specimen. Since the angle and resolution of the two-dimensional cross-sectional images differed for each specimen, because each sample could not perfectly align with one other while scanning, all of the obtained two-dimensional cross-sectional images were subjected to image processing for reorientation and resizing of the images. The angle was rotated so that the tilt became 0 degrees, and the resolution was adjusted to the lowest resolution of the specimens acquired in this study so that the number of pixels per 1 mm was 13. Then, the surrounding area was trimmed so that only the aluminum foam part of the image was included. This process was performed on all images of the specimens. Figure 2c shows an enlarged image of Figure 2b after image processing.

Compression tests of the prepared specimens were performed using a universal testing machine (Instron 5582). The compression speed was 5 mm/min. The compressive stress *σ* was estimated by dividing the compressive load obtained from the compression test by the initial cross-sectional area of the specimen (25 mm × 25 mm). The compressive strain *ε* was estimated by dividing the compressive displacement obtained from the compression test by the initial height of a specimen (25 mm). Plateau stress was defined as the average *σ* at *ε* = 20–30% on the basis of JIS-H-7902 [45]. The compressive deformation behavior of the specimen during the compression test was recorded using a digital video camera.

Convolutional neural networks were used for machine learning using Wolfram Mathematica (Ver. 12.3). First, the training dataset was trained using all of the images associated with plateau stress of the training dataset obtained from the 14 specimens. There were three sets of convolution and pooling layers, followed by a smoothing layer and a linear layer for training. The convolution layers with kernels of size 3 × 3 and a stride of 1 were used. The pooling layers with kernels of size 2 × 2 were used. The loss function was used for the optimization. The “NetChain” built-in symbol was used to specify a neural net. Next, the plateau stress of each specimen was estimated from the images of the test dataset of each specimen. In this study, 300 images of each specimen were randomly selected among which 290 images were used as the training dataset and 10 images were used as the test dataset. Namely, the training was performed using all images of 14 specimens × 290 images. The plateau stress was then estimated from each of the 10 images of the test dataset for one specimen, and the average of the 10 estimated plateau stresses was defined as the estimated plateau stress for that specimen. This process was carried out on each of the 14 specimens.

## 3. Results and Discussion

**Figure 3:**
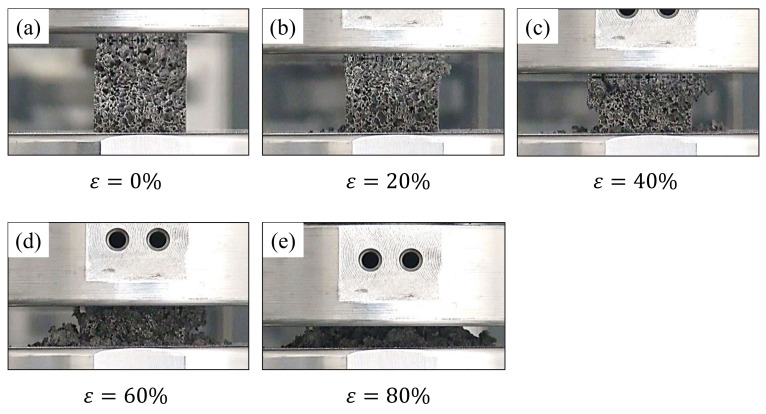
Compressive deformation behavior of prepared aluminum foam.

Figure 3 shows the compressive deformation behavior of a representative specimen. The base material, ADC12 aluminum alloy, was brittle due to its eutectic nature [9,55], and the aluminum foam in this study was also deformed in a brittle manner. That is, scattered fragments were observed during the deformation, as shown in Figure 3b–e, which is consistent with the findings of previous studies [56,57,58,59].

**Figure 4:**
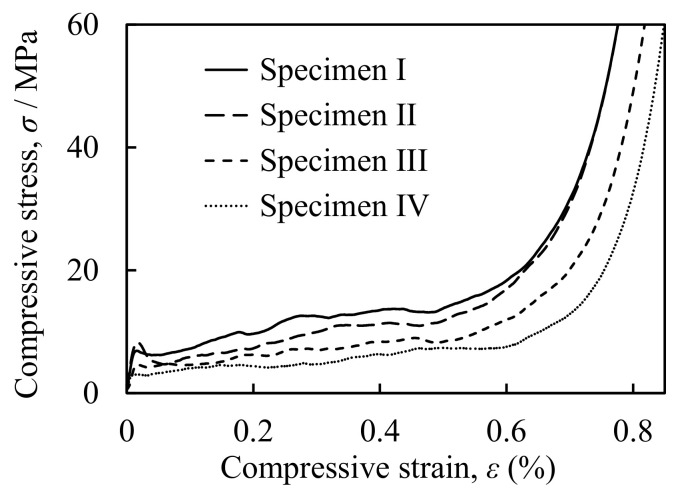
Representative stress–strain curves during compression tests of four specimens.

Figure 4 shows *σ*–*ε* curves for four representative specimens. They have different porosities, and therefore different compressive strengths. Specimens I–IV are shown with different compressive strengths of aluminum foam. That is, Specimen I shows plateau stress of 10.74 MPa and porosity of 76.1%. Specimen II shows plateau stress of 8.09 MPa and porosity of 77.6%. Specimen III shows plateau stress of 6.57 MPa and porosity of 79.9%. Specimen IV shows plateau stress of 4.41 MPa and porosity of 81.7%. First, there is an elastic region in which *σ* increases with *ε*, then a plateau region appears where *σ* remains relatively constant in a wide range of *ε*. Finally, a densification region appears in which *σ* increases rapidly. This trend is similar to those of other aluminum foam specimens [13].

**Figure 5:**
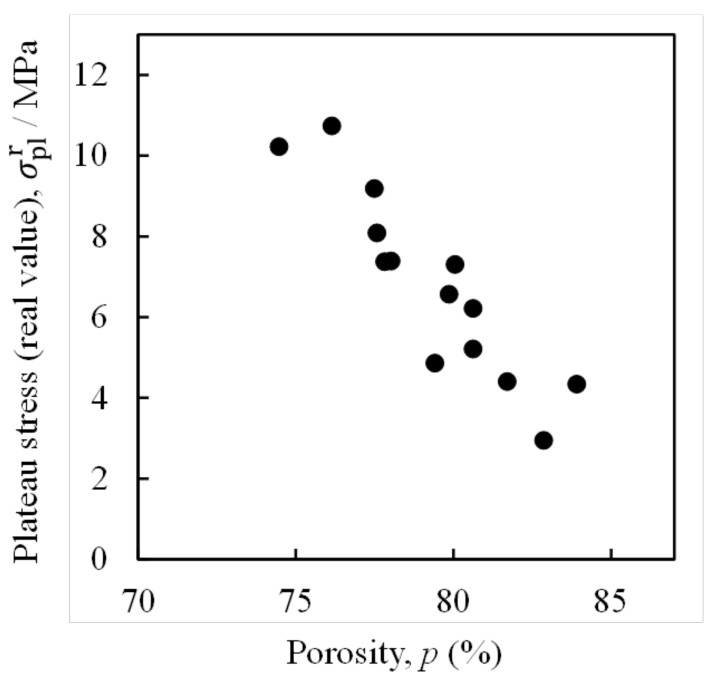
Relationship between porosity *p* and plateau stress $\sigma_{\mathrm{pl}}^{r}$ of prepared aluminum foam.

Figure 5 shows the relationship between the specimen porosity *p* and the plateau stress $\sigma_{\mathrm{pl}}^{r}$ actually obtained from the compression tests of all the 14 specimens tested in this study. *p* was obtained by calculating $p=\left(\rho_{i}-\rho_{f}\right)/\rho_{i}$, where $\rho_{i}$ is the density of the ADC12 aluminum alloy [54] and $\rho_{f}$ is the density of the aluminum foam specimen. The density of the aluminum foam specimen was obtained from the weight and dimensions of the specimen. It can be seen that as *p* increases, $\sigma_{\mathrm{pl}}^{r}$ tends to decrease. However, there is some variation in $\sigma_{\mathrm{pl}}^{r}$. Therefore, it is difficult to estimate $\sigma_{\mathrm{pl}}^{r}$ accurately using only *p*.

**Figure 6:**
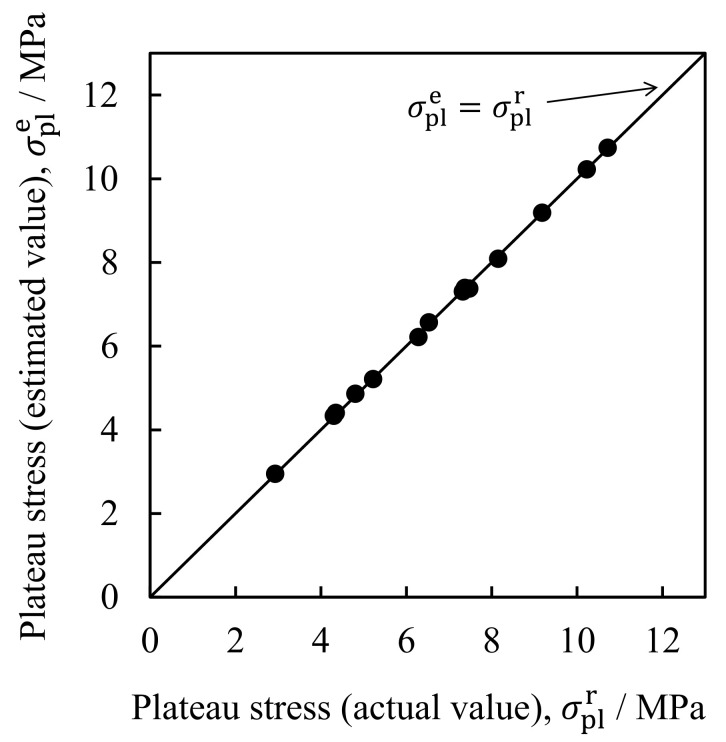
Results of plateau stress estimation. Relationship between actual plateau stress $\sigma_{\mathrm{pl}}^{r}$ obtained from actual compression test and plateau stress $\sigma_{\mathrm{pl}}^{e}$ estimated by machine learning.

Figure 6 shows the results of the plateau stress estimation. The horizontal axis is the actual plateau stress $\sigma_{\mathrm{pl}}^{r}$ obtained from the actual compression test and the vertical axis is the plateau stress $\sigma_{\mathrm{pl}}^{e}$ estimated by machine learning. The straight line in the graph shows the relationship $\sigma_{\mathrm{pl}}^{e}=\sigma_{\mathrm{pl}}^{r}$. It was shown that the estimated values of $\sigma_{\mathrm{pl}}^{e}$ were almost identical to those of $\sigma_{\mathrm{pl}}^{r}$ in the actual compression test, with a correlation coefficient of 0.9998. Therefore, it is considered that the plateau stress can be estimated by training using two-dimensional cross-sectional X-ray CT images of aluminum foam. Note that $\sigma_{\mathrm{pl}}^{e}$ is the average of the plateau stresses estimated from each of the 10 images in the test dataset. For example, when the actual plateau stress is $\sigma_{\mathrm{pl}}^{r}$ = 7.39 MPa, the estimated plateau stress is $\sigma_{\mathrm{pl}}^{e}$ = 7.37 MPa, where the plateau stress estimated from each image ranges from 6.74 MPa to 7.88 MPa, indicating some variations. Similar variations can be seen in other specimens. However, by taking the average of the plateau stresses estimated from each image, it is possible to evaluate the overall trend of the plateau stress of specimens.

## 4. Conclusions

In this study, we attempted to estimate the plateau stress of aluminum foam fabricated by the molten metal foaming method by machine learning using X-ray CT images of aluminum foam. The plateau stresses estimated by machine learning and those actually obtained by the compression test were almost identical. Consequently, it was shown that the plateau stress can be estimated by training using the two-dimensional cross-sectional images obtained nondestructively by X-ray CT imaging. However, the plateau stress estimated from each two-dimensional cross-sectional image varied slightly, and it is expected that the trend of the plateau stress of the entire specimen can be evaluated by taking the average plateau stress estimated from each image.

## Data Availability

Not applicable.

## References

[1] Banhart J. (2001). Manufacture, characterisation and application of cellular metals and metal foams. Prog. Mater. Sci..

[2] García-Moreno F. (2016). Commercial Applications of Metal Foams: Their Properties and Production. Materials.

[3] Duarte I., Vesenjak M., Vide M.J. (2019). Automated Continuous Production Line of Parts Made of Metallic Foams. Metals.

[4] Wan T., Liu Y., Zhou C., Chen X., Li Y. (2021). Fabrication, properties, and applications of open-cell aluminum foams: A review. J. Mater. Sci. Technol..

[5] Zhang J., An Y., Ma H. (2022). Research Progress in the Preparation of Aluminum Foam Composite Structures. Metals.

[6] Maire E., Fazekas A., Salvo L., Dendievel R., Youssef S., Cloetens P., Letang J.M. (2003). X-ray tomography applied to the characterization of cellular materials. Related finite element modeling problems. Compos. Sci. Technol..

[7] Moreno F.G., Fromme M., Banhart J. (2004). Real-time X-ray Radioscopy on Metallic Foams Using a Compact Micro-Focus Source. Adv. Eng. Mater..

[8] Garcia-Moreno F., Mukherjee M., Jiménez C., Rack A., Banhart J. (2011). Metal Foaming Investigated by X-ray Radioscopy. Metals.

[9] Hangai Y., Takahashi K., Yamaguchi R., Utsunomiya T., Kitahara S., Kuwazuru O., Yoshikawa N. (2012). Nondestructive observation of pore structure deformation behavior of functionally graded aluminum foam by X-ray computed tomography. Mater. Sci. Eng. A.

[10] García-Moreno F., Kamm P.H., Neu T.R., Bülk F., Mokso R., Schlepütz C.M., Stampanoni M., Banhart J. (2019). Using X-ray tomoscopy to explore the dynamics of foaming metal. Nat. Commun..

[11] Hangai Y., Kawato D., Ando M., Ohashi M., Morisada Y., Ogura T., Fujii H., Nagahiro R., Amagai K., Utsunomiya T. (2020). Nondestructive observation of pores during press forming of aluminum foam by X-ray radiography. Mater. Charact..

[12] Hangai Y., Kawato D., Ohashi M., Ando M., Ogura T., Morisada Y., Fujii H., Kamakoshi Y., Mitsugi H., Amagai K. (2021). X-ray Radiography Inspection of Pores of Thin Aluminum Foam during Press Forming Immediately after Foaming. Metals.

[13] Gibson L.J. (2000). Mechanical Behavior of Metallic Foams. Annu. Rev. Mater. Sci..

[14] Goodall R., Marmottant A., Salvo L., Mortensen A. (2007). Spherical pore replicated microcellular aluminium: Processing and influence on properties. Mater. Sci. Eng. A.

[15] Bafti H., Habibolahzadeh A. (2013). Compressive properties of aluminum foam produced by powder-Carbamide spacer route. Mater. Des..

[16] Al-Ketan O., Rowshan R., Abu Al-Rub R.K. (2018). Topology-mechanical property relationship of 3D printed strut, skeletal, and sheet based periodic metallic cellular materials. Addit. Manuf..

[17] Orbulov I.N., Szlancsik A., Kemény A., Kincses D. (2020). Compressive mechanical properties of low-cost, aluminium matrix syntactic foams. Compos. Part A Appl. Sci. Manuf..

[18] Guo S., Yue X., Kitazono K. (2021). Anisotropic Compression Behavior of Additively Manufactured Porous Titanium with Ordered Open-Cell Structures at Different Temperatures. Mater. Trans..

[19] Liu X., Wada T., Suzuki A., Takata N., Kobashi M., Kato M. (2020). Understanding and suppressing shear band formation in strut-based lattice structures manufactured by laser powder bed fusion. Mater. Des..

[20] Ryu K.M., An J.Y., Cho W.-S., Yoo Y.-C., Kim H.S. (2005). Mechanical Modeling of Al-Mg Alloy Open-Cell Foams. Mater. Trans..

[21] Wang J., Wang N., Liu X., Ding J., Xia X., Chen X., Zhao W. (2018). Compressive Deformation Behavior of Closed-Cell Micro-Pore Magnesium Composite Foam. Materials.

[22] Skibinski J., Cwieka K., Ibrahim S.H., Wejrzanowski T. (2019). Influence of Pore Size Variation on Thermal Conductivity of Open-Porous Foams. Materials.

[23] Belardi V., Fanelli P., Trupiano S., Vivio F. (2021). Multiscale analysis and mechanical characterization of open-cell foams by simplified FE modeling. Eur. J. Mech. A Solids.

[24] Ohgaki T., Toda H., Kobayashi M., Uesugi K., Kobayashi T., Niinomi M., Akahori T., Makii K., Aruga Y. (2006). In-situ High-resolution X-ray CT Observation of Compressive and Damage Behaviour of Aluminium Foams by Local Tomography Technique. Adv. Eng. Mater..

[25] Jeon I., Asahina T., Kang K.-J., Im S., Lu T.J. (2010). Finite element simulation of the plastic collapse of closed-cell aluminum foams with X-ray computed tomography. Mech. Mater..

[26] Michailidis N., Stergioudi F., Omar H., Tsipas D. (2010). FEM modeling of the response of porous Al in compression. Comput. Mater. Sci..

[27] Hangai Y., Yamaguchi R., Takahashi S., Utsunomiya T., Kuwazuru O., Yoshikawa N. (2012). Deformation Behavior Estimation of Aluminum Foam by X-ray CT Image-based Finite Element Analysis. Met. Mater. Trans. A.

[28] Fiedler T., Sulong M.A., Mathier V., Belova I., Younger C., Murch G. (2014). Mechanical properties of aluminium foam derived from infiltration casting of salt dough. Comput. Mater. Sci..

[29] Duarte I., Fiedler T., Krstulović-Opara L., Vesenjak M. (2020). Brief Review on Experimental and Computational Techniques for Characterization of Cellular Metals. Metals.

[30] Heitor D., Duarte I., Dias-De-Oliveira J. (2021). Aluminium Alloy Foam Modelling and Prediction of Elastic Properties Using X-ray Microcomputed Tomography. Metals.

[31] Peng C., Liu C., Liao Z., Yang B., Tang L., Yang L., Jiang Z. (2022). Automatic 3D image based finite element modelling for metallic foams and accuracy verification of digital volume correlation. Int. J. Mech. Sci..

[32] Yaseen Z.M., Deo R.C., Hilal A., Abd A.M., Bueno L.C., Salcedo-Sanz S., Nehdi M.L. (2018). Predicting compressive strength of lightweight foamed concrete using extreme learning machine model. Adv. Eng. Softw..

[33] Nguyen T., Kashani A., Ngo T., Bordas S. (2018). Deep neural network with high-order neuron for the prediction of foamed concrete strength. Comput. Civ. Infrastruct. Eng..

[34] Dudzik M., Stręk A.M. (2020). ANN Architecture Specifications for Modelling of Open-Cell Aluminum under Compression. Math. Probl. Eng..

[35] Avalos-Gauna E., Zhao Y., Palafox L., Ortiz-Monasterio-Martínez P. (2021). Porous Metal Properties Analysis: A Machine Learning Approach. JOM.

[36] E Rodríguez-Sánchez A., Plascencia-Mora H. (2021). A machine learning approach to estimate the strain energy absorption in expanded polystyrene foams. J. Cell. Plast..

[37] Ullah H.S., Khushnood R.A., Farooq F., Ahmad J., Vatin N.I., Ewais D.Y.Z. (2022). Prediction of Compressive Strength of Sustainable Foam Concrete Using Individual and Ensemble Machine Learning Approaches. Materials.

[38] Dargan S., Kumar M., Ayyagari M.R., Kumar G. (2020). A Survey of Deep Learning and Its Applications: A New Paradigm to Machine Learning. Arch. Comput. Methods Eng..

[39] Gibson G.M., Johnson S.D., Padgett M.J. (2020). Single-pixel imaging 12 years on: A review. Opt. Express.

[40] Dong S., Wang P., Abbas K. (2021). A survey on deep learning and its applications. Comput. Sci. Rev..

[41] Sun H., Burton H.V., Huang H. (2020). Machine learning applications for building structural design and performance assessment: State-of-the-art review. J. Build. Eng..

[42] Abdar M., Pourpanah F., Hussain S., Rezazadegan D., Liu L., Ghavamzadeh M., Fieguth P., Cao X., Khosravi A., Acharya U.R. (2021). A review of uncertainty quantification in deep learning: Techniques, applications and challenges. Inf. Fusion.

[43] Alzubaidi L., Zhang J., Humaidi A.J., Al-Dujaili A., Duan Y., Al-Shamma O., Santamaría J., Fadhel M.A., Al-Amidie M., Farhan L. (2021). Review of deep learning: Concepts, CNN architectures, challenges, applications, future directions. J. Big Data.

[44] Hangai Y., Okada K., Tanaka Y., Matsuura T., Amagai K., Suzuki R., Nakazawa N. (2022). Classification of Mechanical Properties of Aluminum Foam by Machine Learning. Mater. Trans..

[45] (2016). Method for Compressive Test of Porous Metals.

[46] Miyoshi T., Itoh M., Akiyama S., Kitahara A. (2000). Alporas aluminum foam: Production process, properties, and applications. Adv. Eng. Mater..

[47] Kadoi K., Babcsán N., Nakae H. (2009). Heat Treatment of TiH2 Powder to Control Decomposition Phenomenon for Aluminum Foam Fabrication by Melt Route. Mater. Trans..

[48] Zhang B., Hu S., Fan Z. (2018). Anisotropic Compressive Behavior of Functionally Density Graded Aluminum Foam Prepared by Controlled Melt Foaming Process. Materials.

[49] Kuwahara T., Osaka T., Saito M., Suzuki S. (2019). Compressive Properties of A2024 Alloy Foam Fabricated through a Melt Route and a Semi-Solid Route. Metals.

[50] Takamatsu S., Kuwahara T., Kochi R., Suzuki S. (2020). Percolation of Primary Crystals in Cell Walls of Aluminum Alloy Foam via Semi-Solid Route. Metals.

[51] Byakova A., Gnyloskurenko S., Vlasov A., Yevych Y., Semenov N. (2022). The Mechanical Performance of Aluminum Foam Fabricated by Melt Processing with Different Foaming Agents: A Comparative Analysis. Metals.

[52] Parveez B., Jamal N.A., Anuar H., Ahmad Y., Aabid A., Baig M. (2022). Microstructure and Mechanical Properties of Metal Foams Fabricated via Melt Foaming and Powder Metallurgy Technique: A Review. Materials.

[53] Yamamoto T., Hangai Y., Mitsugi H., Koyama S., Suzuki R., Matsubara M., Fujii H. (2022). Fabrication of porous aluminum composites containing hollow ceramics. J. Porous Mater..

[54] The Japan Institute of Light Metals (1991). Structures and Properties of Aluminum.

[55] Okayasu M., Ohkura Y., Takeuchi S., Takasu S., Ohfuji H., Shiraishi T. (2012). A study of the mechanical properties of an Al–Si–Cu alloy (ADC12) produced by various casting processes. Mater. Sci. Eng. A.

[56] Hangai Y., Takahashi K., Utsunomiya T., Kitahara S., Kuwazuru O., Yoshikawa N. (2012). Fabrication of functionally graded aluminum foam using aluminum alloy die castings by friction stir processing. Mater. Sci. Eng. A.

[57] Utsunomiya T., Kubota N., Hangai Y., Ishima T., Kawashima H., Kuwazuru O., Yoshikawa N. (2014). High-speed compressive properties of porous material fabricated using jis adc12 aluminum alloy die castings. J. Jpn. Foundry Eng. Soc..

[58] Hangai Y., Nakano Y., Utsunomiya T., Kuwazuru O., Yoshikawa N. (2017). Drop Weight Impact Behavior of Al-Si-Cu Alloy Foam-Filled Thin-Walled Steel Pipe Fabricated by Friction Stir Back Extrusion. J. Mater. Eng. Perform..

[59] Hangai Y., Utsunomiya T., Kuwazuru O., Kitahara S., Yoshikawa N. (2015). Deformation and Plateau Region of Functionally Graded Aluminum Foam by Amount Combinations of Added Blowing Agent. Materials.

